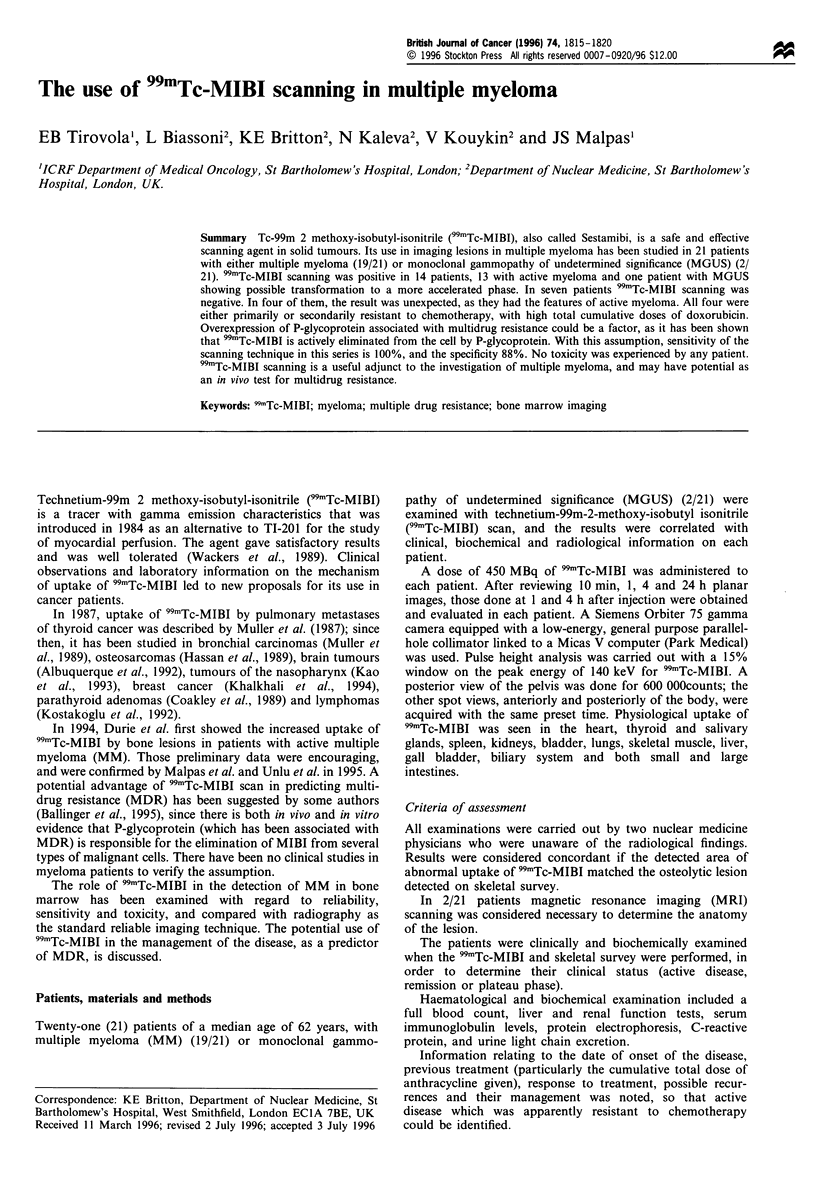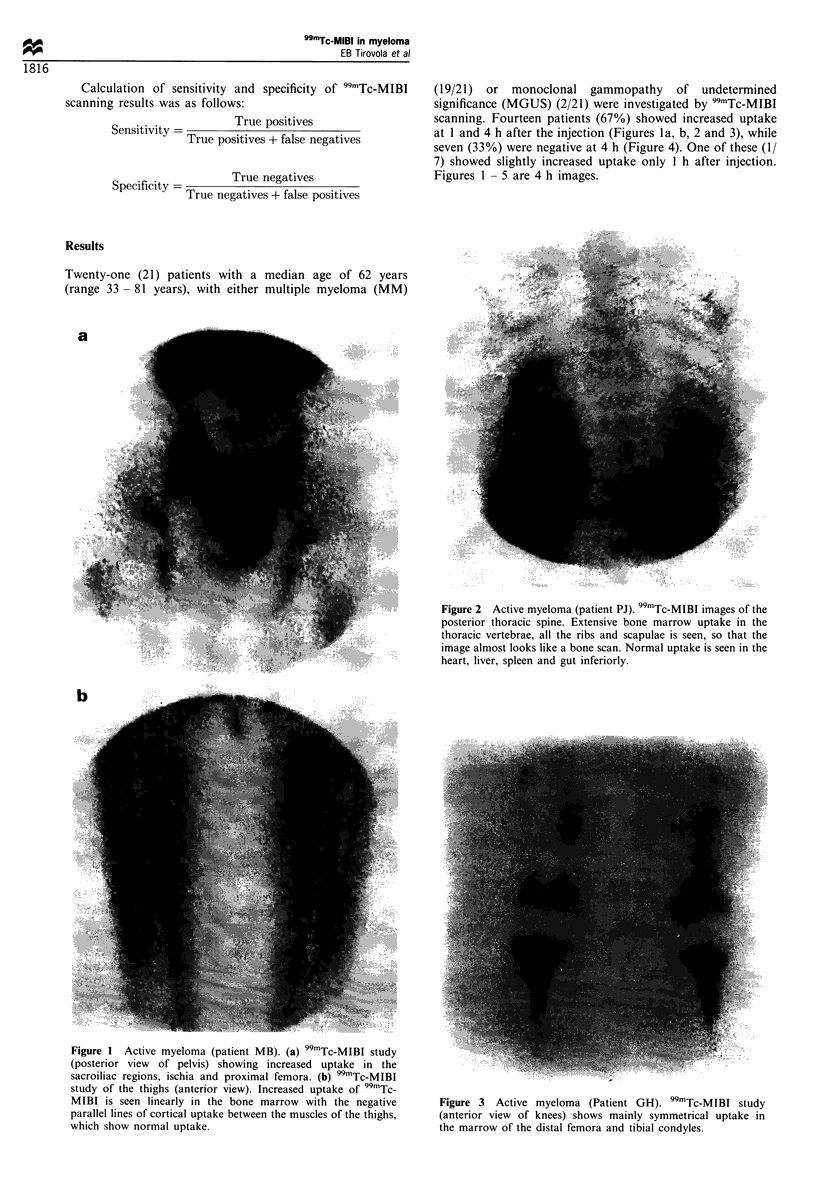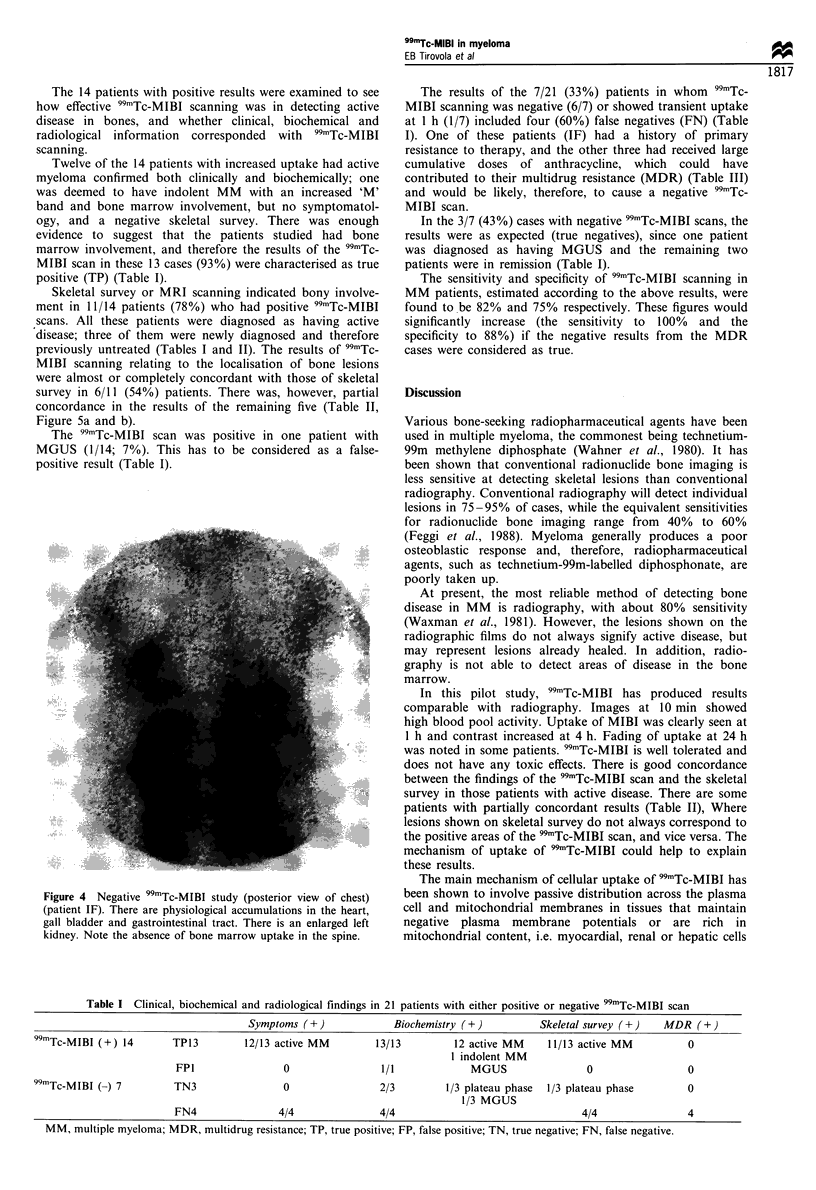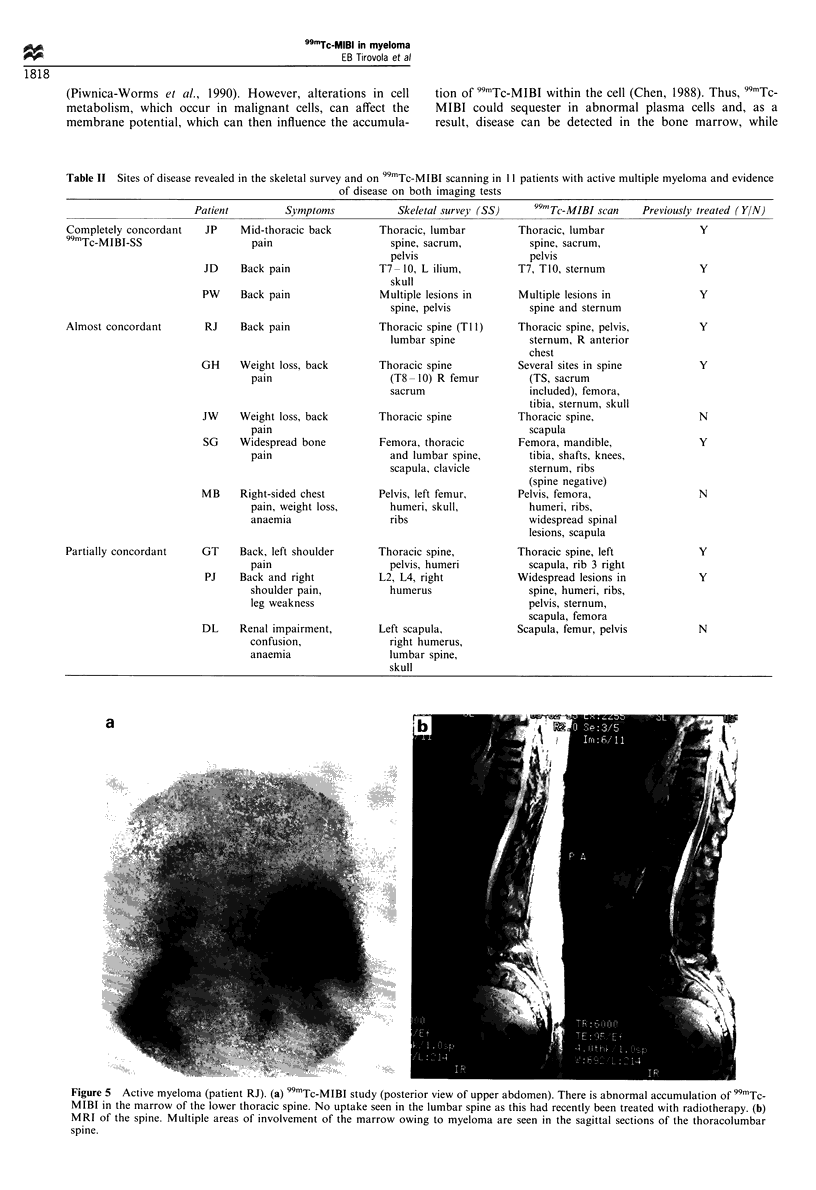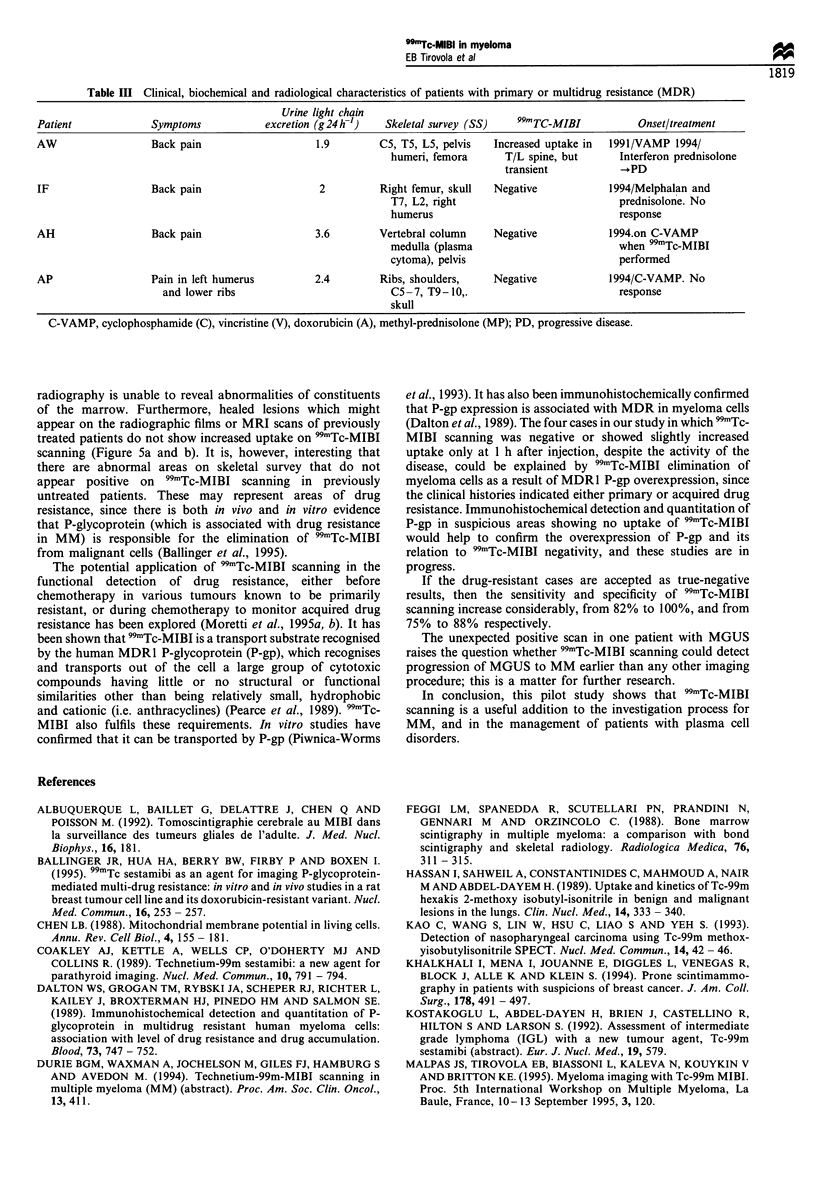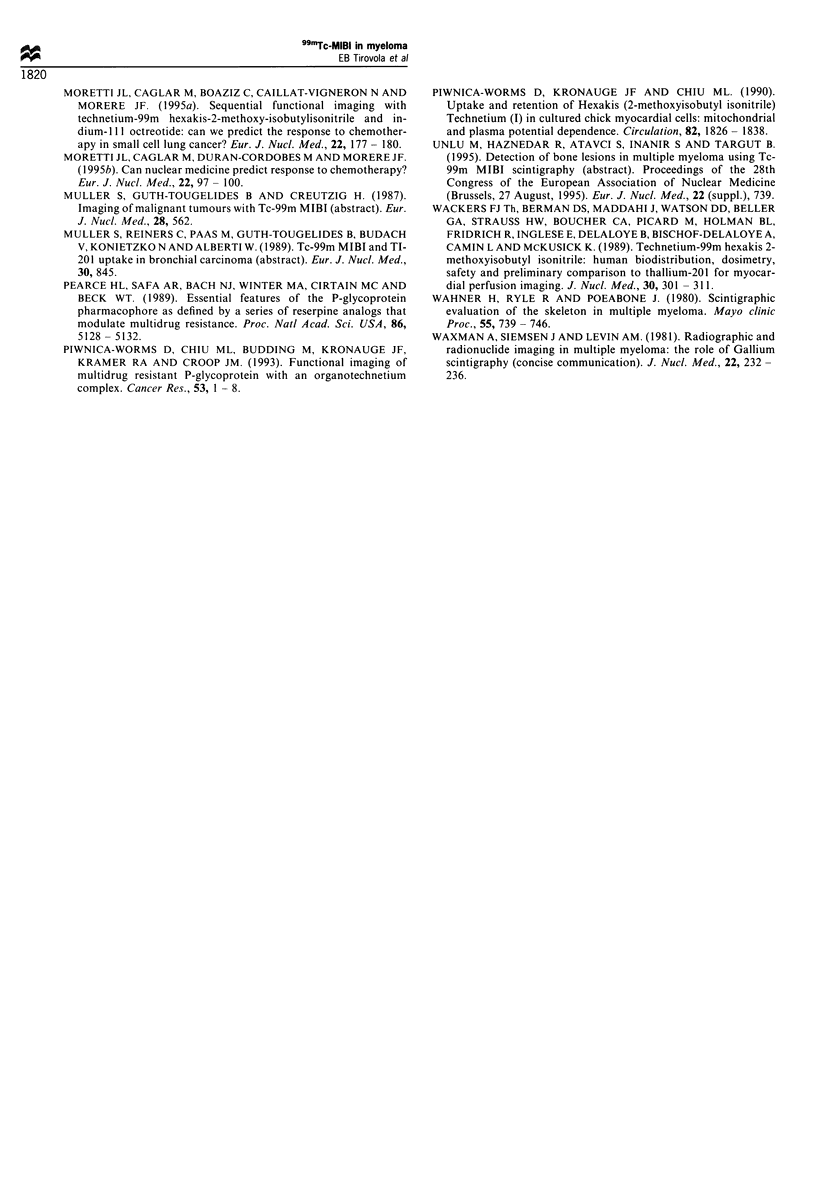# The use of 99mTc-MIBI scanning in multiple myeloma.

**DOI:** 10.1038/bjc.1996.636

**Published:** 1996-12

**Authors:** E. B. Tirovola, L. Biassoni, K. E. Britton, N. Kaleva, V. Kouykin, J. S. Malpas

**Affiliations:** ICRF Department of Medical Oncology, St Bartholomew's Hospital, London, UK.

## Abstract

**Images:**


British Journal of Cancer (1996) 74, 1815-1820
© 1996 Stockton Press All rights reserved 0007-0920/96 $12.00

### The use of $^{99m}$Tc-MIBI scanning in multiple myeloma

EB Tirovola[1], L Biassoni[2], KE Britton[2], N Kaleva[2], V Kouykin[2] and JS Malpas[1]

*[1]ICRF Department of Medical Oncology, St Bartholomew's Hospital, London; [2]Department of Nuclear Medicine, St Bartholomew's Hospital, London, UK.*

**Summary** Tc-99m 2 methoxy-isobutyl-isonitrile ($^{99m}$Tc-MIBI), also called Sestamibi, is a safe and effective scanning agent in solid tumours. Its use in imaging lesions in multiple myeloma has been studied in 21 patients with either multiple myeloma (19/21) or monoclonal gammopathy of undetermined significance (MGUS) (2/21). $^{99m}$Tc-MIBI scanning was positive in 14 patients, 13 with active myeloma and one patient with MGUS showing possible transformation to a more accelerated phase. In seven patients $^{99m}$Tc-MIBI scanning was negative. In four of them, the result was unexpected, as they had the features of active myeloma. All four were either primarily or secondarily resistant to chemotherapy, with high total cumulative doses of doxorubicin. Overexpression of P-glycoprotein associated with multidrug resistance could be a factor, as it has been shown that $^{99m}$Tc-MIBI is actively eliminated from the cell by P-glycoprotein. With this assumption, sensitivity of the scanning technique in this series is 100%, and the specificity 88%. No toxicity was experienced by any patient. $^{99m}$Tc-MIBI scanning is a useful adjunct to the investigation of multiple myeloma, and may have potential as an *in vivo* test for multidrug resistance.

**Keywords:** $^{99m}$Tc-MIBI; myeloma; multiple drug resistance; bone marrow imaging

Technetium-99m 2 methoxy-isobutyl-isonitrile ($^{99m}$Tc-MIBI) is a tracer with gamma emission characteristics that was introduced in 1984 as an alternative to TI-201 for the study of myocardial perfusion. The agent gave satisfactory results and was well tolerated (Wackers *et al.*, 1989). Clinical observations and laboratory information on the mechanism of uptake of $^{99m}$Tc-MIBI led to new proposals for its use in cancer patients.

In 1987, uptake of $^{99m}$Tc-MIBI by pulmonary metastases of thyroid cancer was described by Muller *et al.* (1987); since then, it has been studied in bronchial carcinomas (Muller *et al.*, 1989), osteosarcomas (Hassan *et al.*, 1989), brain tumours (Albuquerque *et al.*, 1992), tumours of the nasopharynx (Kao *et al.*, 1993), breast cancer (Khalkhali *et al.*, 1994), parathyroid adenomas (Coakley *et al.*, 1989) and lymphomas (Kostakóglu *et al.*, 1992).

In 1994, Durie *et al.* first showed the increased uptake of $^{99m}$Tc-MIBI by bone lesions in patients with active multiple myeloma (MM). Those preliminary data were encouraging, and were confirmed by Malpas *et al.* and Unlu *et al.* in 1995. A potential advantage of $^{99m}$Tc-MIBI scan in predicting multidrug resistance (MDR) has been suggested by some authors (Ballinger *et al.*, 1995), since there is both *in vivo* and *in vitro* evidence that P-glycoprotein (which has been associated with MDR) is responsible for the elimination of MIBI from several types of malignant cells. There have been no clinical studies in myeloma patients to verify the assumption.

The role of $^{99m}$Tc-MIBI in the detection of MM in bone marrow has been examined with regard to reliability, sensitivity and toxicity, and compared with radiography as the standard reliable imaging technique. The potential use of $^{99m}$Tc-MIBI in the management of the disease, as a predictor of MDR, is discussed.

### Patients, materials and methods

Twenty-one (21) patients of a median age of 62 years, with multiple myeloma (MM) (19/21) or monoclonal gammopathy of undetermined significance (MGUS) (2/21) were examined with technetium-99m-2-methoxy-isobutyl isonitrile ($^{99m}$Tc-MIBI) scan, and the results were correlated with clinical, biochemical and radiological information on each patient.

A dose of 450 MBq of $^{99m}$Tc-MIBI was administered to each patient. After reviewing 10 min, 1, 4 and 24 h planar images, those done at 1 and 4 h after injection were obtained and evaluated in each patient. A Siemens Orbiter 75 gamma camera equipped with a low-energy, general purpose parallel-hole collimator linked to a Micas V computer (Park Medical) was used. Pulse height analysis was carried out with a 15% window on the peak energy of 140 keV for $^{99m}$Tc-MIBI. A posterior view of the pelvis was done for 600 000counts; the other spot views, anteriorly and posteriorly of the body, were acquired with the same preset time. Physiological uptake of $^{99m}$Tc-MIBI was seen in the heart, thyroid and salivary glands, spleen, kidneys, bladder, lungs, skeletal muscle, liver, gall bladder, biliary system and both small and large intestines.

### *Criteria of assessment*

All examinations were carried out by two nuclear medicine physicians who were unaware of the radiological findings. Results were considered concordant if the detected area of abnormal uptake of $^{99m}$Tc-MIBI matched the osteolytic lesion detected on skeletal survey.

In 2/21 patients magnetic resonance imaging (MRI) scanning was considered necessary to determine the anatomy of the lesion.

The patients were clinically and biochemically examined when the $^{99m}$Tc-MIBI and skeletal survey were performed, in order to determine their clinical status (active disease, remission or plateau phase).

Haematological and biochemical examination included a full blood count, liver and renal function tests, serum immunoglobulin levels, protein electrophoresis, C-reactive protein, and urine light chain excretion.

Information relating to the date of onset of the disease, previous treatment (particularly the cumulative total dose of anthracycline given), response to treatment, possible recurrences and their management was noted, so that active disease which was apparently resistant to chemotherapy could be identified.

Correspondence: KE Britton, Department of Nuclear Medicine, St Bartholomew's Hospital, West Smithfield, London EC1A 7BE, UK
Received 11 March 1996; revised 2 July 1996; accepted 3 July 1996

Calculation of sensitivity and specificity of $^{99m}$Tc-MIBI scanning results was as follows:

$$\text{Sensitivity} = \frac{\text{True positives}}{\text{True positives} + \text{false negatives}}$$

$$\text{Specificity} = \frac{\text{True negatives}}{\text{True negatives} + \text{false positives}}$$

### Results

Twenty-one (21) patients with a median age of 62 years (range 33 – 81 years), with either multiple myeloma (MM) (19/21) or monoclonal gammopathy of undetermined significance (MGUS) (2/21) were investigated by $^{99m}$Tc-MIBI scanning. Fourteen patients (67%) showed increased uptake at 1 and 4 h after the injection (Figures 1a, b, 2 and 3), while seven (33%) were negative at 4 h (Figure 4). One of these (1/7) showed slightly increased uptake only 1 h after injection. Figures 1 – 5 are 4 h images.

**Figure 1** Active myeloma (patient MB). (**a**) $^{99m}$Tc-MIBI study (posterior view of pelvis) showing increased uptake in the sacroiliac regions, ischia and proximal femora. (**b**) $^{99m}$Tc-MIBI study of the thighs (anterior view). Increased uptake of $^{99m}$Tc-MIBI is seen linearly in the bone marrow with the negative parallel lines of cortical uptake between the muscles of the thighs, which show normal uptake.

**Figure 2** Active myeloma (patient PJ). $^{99m}$Tc-MIBI images of the posterior thoracic spine. Extensive bone marrow uptake in the thoracic vertebrae, all the ribs and scapulae is seen, so that the image almost looks like a bone scan. Normal uptake is seen in the heart, liver, spleen and gut inferiorly.

**Figure 3** Active myeloma (Patient GH). $^{99m}$Tc-MIBI study (anterior view of knees) shows mainly symmetrical uptake in the marrow of the distal femora and tibial condyles.

The 14 patients with positive results were examined to see how effective $^{99m}$Tc-MIBI scanning was in detecting active disease in bones, and whether clinical, biochemical and radiological information corresponded with $^{99m}$Tc-MIBI scanning.

Twelve of the 14 patients with increased uptake had active myeloma confirmed both clinically and biochemically; one was deemed to have indolent MM with an increased 'M' band and bone marrow involvement, but no symptomatology, and a negative skeletal survey. There was enough evidence to suggest that the patients studied had bone marrow involvement, and therefore the results of the $^{99m}$Tc-MIBI scan in these 13 cases (93%) were characterised as true positive (TP) (Table I).

Skeletal survey or MRI scanning indicated bony involvement in 11/14 patients (78%) who had positive $^{99m}$Tc-MIBI scans. All these patients were diagnosed as having active disease; three of them were newly diagnosed and therefore previously untreated (Tables I and II). The results of $^{99m}$Tc-MIBI scanning relating to the localisation of bone lesions were almost or completely concordant with those of skeletal survey in 6/11 (54%) patients. There was, however, partial concordance in the results of the remaining five (Table II, Figure 5a and b).

The $^{99m}$Tc-MIBI scan was positive in one patient with MGUS (1/14; 7%). This has to be considered as a false-positive result (Table I).

**Figure 4** Negative $^{99m}$Tc-MIBI study (posterior view of chest) (patient IF). There are physiological accumulations in the heart, gall bladder and gastrointestinal tract. There is an enlarged left kidney. Note the absence of bone marrow uptake in the spine.

The results of the 7/21 (33%) patients in whom $^{99m}$Tc-MIBI scanning was negative (6/7) or showed transient uptake at 1 h (1/7) included four (60%) false negatives (FN) (Table I). One of these patients (IF) had a history of primary resistance to therapy, and the other three had received large cumulative doses of anthracycline, which could have contributed to their multidrug resistance (MDR) (Table III) and would be likely, therefore, to cause a negative $^{99m}$Tc-MIBI scan.

In the 3/7 (43%) cases with negative $^{99m}$Tc-MIBI scans, the results were as expected (true negatives), since one patient was diagnosed as having MGUS and the remaining two patients were in remission (Table I).

The sensitivity and specificity of $^{99m}$Tc-MIBI scanning in MM patients, estimated according to the above results, were found to be 82% and 75% respectively. These figures would significantly increase (the sensitivity to 100% and the specificity to 88%) if the negative results from the MDR cases were considered as true.

### Discussion

Various bone-seeking radiopharmaceutical agents have been used in multiple myeloma, the commonest being technetium-99m methylene diphosphate (Wahner *et al.*, 1980). It has been shown that conventional radionuclide bone imaging is less sensitive at detecting skeletal lesions than conventional radiography. Conventional radiography will detect individual lesions in 75–95% of cases, while the equivalent sensitivities for radionuclide bone imaging range from 40% to 60% (Feggi *et al.*, 1988). Myeloma generally produces a poor osteoblastic response and, therefore, radiopharmaceutical agents, such as technetium-99m-labelled diphosphonate, are poorly taken up.

At present, the most reliable method of detecting bone disease in MM is radiography, with about 80% sensitivity (Waxman *et al.*, 1981). However, the lesions shown on the radiographic films do not always signify active disease, but may represent lesions already healed. In addition, radiography is not able to detect areas of disease in the bone marrow.

In this pilot study, $^{99m}$Tc-MIBI has produced results comparable with radiography. Images at 10 min showed high blood pool activity. Uptake of MIBI was clearly seen at 1 h and contrast increased at 4 h. Fading of uptake at 24 h was noted in some patients. $^{99m}$Tc-MIBI is well tolerated and does not have any toxic effects. There is good concordance between the findings of the $^{99m}$Tc-MIBI scan and the skeletal survey in those patients with active disease. There are some patients with partially concordant results (Table II), Where lesions shown on skeletal survey do not always correspond to the positive areas of the $^{99m}$Tc-MIBI scan, and vice versa. The mechanism of uptake of $^{99m}$Tc-MIBI could help to explain these results.

The main mechanism of cellular uptake of $^{99m}$Tc-MIBI has been shown to involve passive distribution across the plasma cell and mitochondrial membranes in tissues that maintain negative plasma membrane potentials or are rich in mitochondrial content, i.e. myocardial, renal or hepatic cells

**Table I** Clinical, biochemical and radiological findings in 21 patients with either positive or negative $^{99m}$Tc-MIBI scan

| | | *Symptoms (+)* | *Biochemistry (+)* | | *Skeletal survey (+)* | *MDR (+)* |
|---|---|---|---|---|---|---|
| $^{99m}$Tc-MIBI (+) 14 | TP13 | 12/13 active MM | 13/13 | 12 active MM<br>1 indolent MM | 11/13 active MM | 0 |
| | FP1 | 0 | 1/1 | MGUS | 0 | 0 |
| $^{99m}$Tc-MIBI (–) 7 | TN3 | 0 | 2/3 | 1/3 plateau phase<br>1/3 MGUS | 1/3 plateau phase | 0 |
| | FN4 | 4/4 | 4/4 | | 4/4 | 4 |

MM, multiple myeloma; MDR, multidrug resistance; TP, true positive; FP, false positive; TN, true negative; FN, false negative.

(Piwnica-Worms *et al.*, 1990). However, alterations in cell metabolism, which occur in malignant cells, can affect the membrane potential, which can then influence the accumulation of $^{99m}$Tc-MIBI within the cell (Chen, 1988). Thus, $^{99m}$Tc-MIBI could sequester in abnormal plasma cells and, as a result, disease can be detected in the bone marrow, while

**Table II** Sites of disease revealed in the skeletal survey and on $^{99m}$Tc-MIBI scanning in 11 patients with active multiple myeloma and evidence of disease on both imaging tests

| | *Patient* | *Symptoms* | *Skeletal survey (SS)* | *$^{99m}$Tc-MIBI scan* | *Previously treated (Y/N)* |
|---|---|---|---|---|---|
| Completely concordant $^{99m}$Tc-MIBI-SS | JP | Mid-thoracic back pain | Thoracic, lumbar spine, sacrum, pelvis | Thoracic, lumbar spine, sacrum, pelvis | Y |
| | JD | Back pain | T7–10, L ilium, skull | T7, T10, sternum | Y |
| | PW | Back pain | Multiple lesions in spine, pelvis | Multiple lesions in spine and sternum | Y |
| Almost concordant | RJ | Back pain | Thoracic spine (T11) lumbar spine | Thoracic spine, pelvis, sternum, R anterior chest | Y |
| | GH | Weight loss, back pain | Thoracic spine (T8–10) R femur sacrum | Several sites in spine (TS, sacrum included), femora, tibia, sternum, skull | Y |
| | JW | Weight loss, back pain | Thoracic spine | Thoracic spine, scapula | N |
| | SG | Widespread bone pain | Femora, thoracic and lumbar spine, scapula, clavicle | Femora, mandible, tibia, shafts, knees, sternum, ribs (spine negative) | Y |
| | MB | Right-sided chest pain, weight loss, anaemia | Pelvis, left femur, humeri, skull, ribs | Pelvis, femora, humeri, ribs, widespread spinal lesions, scapula | N |
| Partially concordant | GT | Back, left shoulder pain | Thoracic spine, pelvis, humeri | Thoracic spine, left scapula, rib 3 right | Y |
| | PJ | Back and right shoulder pain, leg weakness | L2, L4, right humerus | Widespread lesions in spine, humeri, ribs, pelvis, sternum, scapula, femora | Y |
| | DL | Renal impairment, confusion, anaemia | Left scapula, right humerus, lumbar spine, skull | Scapula, femur, pelvis | N |

**Figure 5** Active myeloma (patient RJ). (**a**) $^{99m}$Tc-MIBI study (posterior view of upper abdomen). There is abnormal accumulation of $^{99m}$Tc-MIBI in the marrow of the lower thoracic spine. No uptake seen in the lumbar spine as this had recently been treated with radiotherapy. (**b**) MRI of the spine. Multiple areas of involvement of the marrow owing to myeloma are seen in the sagittal sections of the thoracolumbar spine.

**Table III** Clinical, biochemical and radiological characteristics of patients with primary or multidrug resistance (MDR)

| *Patient* | *Symptoms* | *Urine light chain excretion (g 24 h$^{-1}$)* | *Skeletal survey (SS)* | *$^{99m}$TC-MIBI* | *Onset/treatment* |
|---|---|---|---|---|---|
| AW | Back pain | 1.9 | C5, T5, L5, pelvis humeri, femora | Increased uptake in T/L spine, but transient | 1991/VAMP 1994/ Interferon prednisolone →PD |
| IF | Back pain | 2 | Right femur, skull T7, L2, right humerus | Negative | 1994/Melphalan and prednisolone. No response |
| AH | Back pain | 3.6 | Vertebral column medulla (plasma cytoma), pelvis | Negative | 1994.on C-VAMP when $^{99m}$Tc-MIBI performed |
| AP | Pain in left humerus and lower ribs | 2.4 | Ribs, shoulders, C5-7, T9-10,. skull | Negative | 1994/C-VAMP. No response |

C-VAMP, cyclophosphamide (C), vincristine (V), doxorubicin (A), methyl-prednisolone (MP); PD, progressive disease.

radiography is unable to reveal abnormalities of constituents of the marrow. Furthermore, healed lesions which might appear on the radiographic films or MRI scans of previously treated patients do not show increased uptake on $^{99m}$Tc-MIBI scanning (Figure 5a and b). It is, however, interesting that there are abnormal areas on skeletal survey that do not appear positive on $^{99m}$Tc-MIBI scanning in previously untreated patients. These may represent areas of drug resistance, since there is both *in vivo* and *in vitro* evidence that P-glycoprotein (which is associated with drug resistance in MM) is responsible for the elimination of $^{99m}$Tc-MIBI from malignant cells (Ballinger *et al.*, 1995).

The potential application of $^{99m}$Tc-MIBI scanning in the functional detection of drug resistance, either before chemotherapy in various tumours known to be primarily resistant, or during chemotherapy to monitor acquired drug resistance has been explored (Moretti *et al.*, 1995*a, b*). It has been shown that $^{99m}$Tc-MIBI is a transport substrate recognised by the human MDR1 P-glycoprotein (P-gp), which recognises and transports out of the cell a large group of cytotoxic compounds having little or no structural or functional similarities other than being relatively small, hydrophobic and cationic (i.e. anthracyclines) (Pearce *et al.*, 1989). $^{99m}$Tc-MIBI also fulfils these requirements. *In vitro* studies have confirmed that it can be transported by P-gp (Piwnica-Worms *et al.*, 1993). It has also been immunohistochemically confirmed that P-gp expression is associated with MDR in myeloma cells (Dalton *et al.*, 1989). The four cases in our study in which $^{99m}$Tc-MIBI scanning was negative or showed slightly increased uptake only at 1 h after injection, despite the activity of the disease, could be explained by $^{99m}$Tc-MIBI elimination of myeloma cells as a result of MDR1 P-gp overexpression, since the clinical histories indicated either primary or acquired drug resistance. Immunohistochemical detection and quantitation of P-gp in suspicious areas showing no uptake of $^{99m}$Tc-MIBI would help to confirm the overexpression of P-gp and its relation to $^{99m}$Tc-MIBI negativity, and these studies are in progress.

If the drug-resistant cases are accepted as true-negative results, then the sensitivity and specificity of $^{99m}$Tc-MIBI scanning increase considerably, from 82% to 100%, and from 75% to 88% respectively.

The unexpected positive scan in one patient with MGUS raises the question whether $^{99m}$Tc-MIBI scanning could detect progression of MGUS to MM earlier than any other imaging procedure; this is a matter for further research.

In conclusion, this pilot study shows that $^{99m}$Tc-MIBI scanning is a useful addition to the investigation process for MM, and in the management of patients with plasma cell disorders.

### References

ALBUQUERQUE L, BAILLET G, DELATTRE J, CHEN Q AND POISSON M. (1992). Tomoscintigraphie cerebrale au MIBI dans la surveillance des tumeurs gliales de l'adulte. *J. Med. Nucl. Biophys.*, **16**, 181.

BALLINGER JR, HUA HA, BERRY BW, FIRBY P AND BOXEN I. (1995). $^{99m}$Tc sestamibi as an agent for imaging P-glycoprotein-mediated multi-drug resistance: *in vitro* and *in vivo* studies in a rat breast tumour cell line and its doxorubicin-resistant variant. *Nucl. Med. Commun.*, **16**, 253 - 257.

CHEN LB. (1988). Mitochondrial membrane potential in living cells. *Annu. Rev. Cell Biol.*, **4**, 155 - 181.

COAKLEY AJ, KETTLE A, WELLS CP, O'DOHERTY MJ AND COLLINS R. (1989). Technetium-99m sestamibi: a new agent for parathyroid imaging. *Nucl. Med. Commun.*, **10**, 791 - 794.

DALTON WS, GROGAN TM, RYBSKI JA, SCHEPER RJ, RICHTER L, KAILEY J, BROXTERMAN HJ, PINEDO HM AND SALMON SE. (1989). Immunohistochemical detection and quantitation of P-glycoprotein in multidrug resistant human myeloma cells: association with level of drug resistance and drug accumulation. *Blood*, **73**, 747 - 752.

DURIE BGM, WAXMAN A, JOCHELSON M, GILES FJ, HAMBURG S AND AVEDON M. (1994). Technetium-99m-MIBI scanning in multiple myeloma (MM) (abstract). *Proc. Am. Soc. Clin. Oncol.*, **13**, 411.

FEGGI LM, SPANEDDA R, SCUTELLARI PN, PRANDINI N, GENNARI M AND ORZINCOLO C. (1988). Bone marrow scintigraphy in multiple myeloma: a comparison with bond scintigraphy and skeletal radiology. *Radiologica Medica*, **76**, 311 - 315.

HASSAN I, SAHWEIL A, CONSTANTINIDES C, MAHMOUD A, NAIR M AND ABDEL-DAYEM H. (1989). Uptake and kinetics of Tc-99m hexakis 2-methoxy isobutyl-isonitrile in benign and malignant lesions in the lungs. *Clin. Nucl. Med.*, **14**, 333 - 340.

KAO C, WANG S, LIN W, HSU C, LIAO S AND YEH S. (1993). Detection of nasopharyngeal carcinoma using Tc-99m methoxy-isobutylisonitrile SPECT. *Nucl. Med. Commun.*, **14**, 42 - 46.

KHALKHALI I, MENA I, JOUANNE E, DIGGLES L, VENEGAS R, BLOCK J, ALLE K AND KLEIN S. (1994). Prone scintimammography in patients with suspicions of breast cancer. *J. Am. Coll. Surg.*, **178**, 491 - 497.

KOSTAKOGLU L, ABDEL-DAYEN H, BRIEN J, CASTELLINO R, HILTON S AND LARSON S. (1992). Assessment of intermediate grade lymphoma (IGL) with a new tumour agent, Tc-99m sestamibi (abstract). *Eur. J. Nucl. Med.*, **19**, 579.

MALPAS JS, TIROVOLA EB, BIASSONI L, KALEVA N, KOUYKIN V AND BRITTON KE. (1995). Myeloma imaging with Tc-99m MIBI. Proc. 5th International Workshop on Multiple Myeloma, La Baule, France, 10-13 September 1995, **3**, 120.

MORETTI JL, CAGLAR M, BOAZIZ C, CAILLAT-VIGNERON N AND MORERE JF. (1995*a*). Sequential functional imaging with technetium-99m hexakis-2-methoxy-isobutylisonitrile and indium-111 octreotide: can we predict the response to chemotherapy in small cell lung cancer? *Eur. J. Nucl. Med.*, **22**, 177 - 180.

MORETTI JL, CAGLAR M, DURAN-CORDOBES M AND MORERE JF. (1995*b*). Can nuclear medicine predict response to chemotherapy? *Eur. J. Nucl. Med.*, **22**, 97 - 100.

MULLER S, GUTH-TOUGELIDES B AND CREUTZIG H. (1987). Imaging of malignant tumours with Tc-99m MIBI (abstract). *Eur. J. Nucl. Med.*, **28**, 562.

MULLER S, REINERS C, PAAS M, GUTH-TOUGELIDES B, BUDACH V, KONIETZKO N AND ALBERTI W. (1989). Tc-99m MIBI and Tl-201 uptake in bronchial carcinoma (abstract). *Eur. J. Nucl. Med.*, **30**, 845.

PEARCE HL, SAFA AR, BACH NJ, WINTER MA, CIRTAIN MC AND BECK WT. (1989). Essential features of the P-glycoprotein pharmacophore as defined by a series of reserpine analogs that modulate multidrug resistance. *Proc. Natl Acad. Sci. USA*, **86**, 5128 - 5132.

PIWNICA-WORMS D, CHIU ML, BUDDING M, KRONAUGE JF, KRAMER RA AND CROOP JM. (1993). Functional imaging of multidrug resistant P-glycoprotein with an organotechnetium complex. *Cancer Res.*, **53**, 1 - 8.

PIWNICA-WORMS D, KRONAUGE JF AND CHIU ML. (1990). Uptake and retention of Hexakis (2-methoxyisobutyl isonitrile) Technetium (I) in cultured chick myocardial cells: mitochondrial and plasma potential dependence. *Circulation*, **82**, 1826 - 1838.

UNLU M, HAZNEDAR R, ATAVCI S, INANIR S AND TARGUT B. (1995). Detection of bone lesions in multiple myeloma using Tc-99m MIBI scintigraphy (abstract). Proceedings of the 28th Congress of the European Association of Nuclear Medicine (Brussels, 27 August, 1995). *Eur. J. Nucl. Med.*, **22** (suppl.), 739.

WACKERS FJ Th, BERMAN DS, MADDAHI J, WATSON DD, BELLER GA, STRAUSS HW, BOUCHER CA, PICARD M, HOLMAN BL, FRIDRICH R, INGLESE E, DELALOYE B, BISCHOF-DELALOYE A, CAMIN L AND MCKUSICK K. (1989). Technetium-99m hexakis 2-methoxyisobutyl isonitrile: human biodistribution, dosimetry, safety and preliminary comparison to thallium-201 for myocardial perfusion imaging. *J. Nucl. Med.*, **30**, 301 - 311.

WAHNER H, RYLE R AND POEABONE J. (1980). Scintigraphic evaluation of the skeleton in multiple myeloma. *Mayo clinic Proc.*, **55**, 739 - 746.

WAXMAN A, SIEMSEN J AND LEVIN AM. (1981). Radiographic and radionuclide imaging in multiple myeloma: the role of Gallium scintigraphy (concise communication). *J. Nucl. Med.*, **22**, 232 - 236.